# Prevalence of Cardiovascular Risk Factors among Tile and Ceramic Workers in Yazd, Iran

**DOI:** 10.5402/2013/921860

**Published:** 2013-12-18

**Authors:** Amir Houshang Mehrparvar, Seyyed Jalil Mirmohammadi, Mehrdad Mostaghaci, Maryam Bahaloo, Mohammad Heydari, Ehsan Samimi, Mahnaz Zohal, Mohammad Hossein Davari

**Affiliations:** ^1^Department of Occupational Medicine, School of Medicine, Shahid Sadoughi University of Medical Sciences, Shahid Rahnemoun Hospital, Farrokhi Avenue, Yazd 89138-14389, Iran; ^2^Industrial Disease Research Center, Shahid Sadoughi University of Medical Sciences, Shahid Rahnemoun Hospital, Farrokhi Avenue, Yazd 89138-14389, Iran; ^3^School of Medicine, Shahid Sadoughi University of Medical Sciences, Shahid Rahnemoun Hospital, Farrokhi Avenue, Yazd 89138-14389, Iran

## Abstract

*Introduction*. Cardiovascular disorders (CVDs) are among the most important diseases in the world and determination of their risk factors is essential for primary and secondary prevention. This study aimed to evaluate these risk factors in workers of tile and ceramic industry, a main industry in Yazd. *Materials and Methods*. In a cross-sectional study, 1075 tile and ceramic workers were selected by simple sampling method. BMI, blood pressure, FBS, and lipid profile were measured and compared to international standards. *Results*. 731 individuals (68%) had at least one risk factor, and 52%, 12%, 3%, and 0.7% had one, two, three, and four risk factors, respectively. The most common risk factor was abnormal BMI (49.6%); low HDL (48.4%) and high TG (14.1%) were in the second and third orders. *Conclusion*. This study showed a relatively high prevalence for CVD risk factors among tile and ceramic workers. Low HDL, high TG, and overweight were the most frequent risk factors in this population.

## 1. Introduction

Cardiovascular disorders (CVDs) are among the most important and frequent chronic and noncommunicable diseases in the world and contribute to 30% of mortalities throughout the world [1]. According to the studies, about 14 million people died due to CVDs in 1990 and it is predicted that this measure reaches 25 million in 2020 [2].

Atherosclerosis and its related factors (risk factors) cause coronary artery disease [3]. These risk factors are classified into two groups: noncorrectable, such as age, race, gender, and correctable, such as smoking, hypercholesterolemia, type II diabetes, hypertension, obesity and overweight, sedentary life style, and stress [4]. Identification of these factors can help us develop practical guidelines which can reduce the incidence of CVDs and their mortality and eventual socioeconomic problems [5].

Physical activity is a predictor of CVDs and their mortality [6]. Nowadays overweight and obesity have created an epidemic with an increased tendency to sedentary life [7] which is the most principal cardiovascular risk factor among industrial workers [8].

It is estimated that worldwide prevalence of hypertension is about one billion and it causes approximately 7.1 million deaths per year [9]. According to World Health Organization (WHO), hypertension accounts for 62% and 49% of cases of CVD and ischemic heart disease, respectively [10].

Other occupational factors which can increase the risk of CVDs include high workload, shift work, noise, and psychosocial factors [11, 12]. Some studies have shown that these risk factors are not assumed as independent, but they may aggravate the effect of other risk factors [13–15].

Some studies have assessed the cardiovascular risk factors among occupational groups. In a study in China, the most prevalent cardiovascular risk factors in steel workers included hypertension, smoking, overweight, and hypercholesterolemia [16]. In another study in Mexico the prevalence of cardiovascular risk factors (hypercholesterolemia, hypertriglyceridemia, overweight, obesity, and hypertension) in males and females older than 30 years was 13.2% and 42.2%, respectively [17]. Among bus drivers, obesity, hypertension, hyperlipidemia, and hyperglycemia were the most frequent cardiovascular risk factors [9].

Evaluation of adults' cardiovascular risk factors is an important issue to reduce the frequency of disorders and their complications. Employees as a large and active population in a society are apt to the risk factors of CVDs some of which may be preventable. So in this study we aimed to evaluate the cardiovascular risk factors of workers of tile and ceramic industry which is the main industry in Yazd. We couldnot find another study conducted on tile and ceramic workers regarding cardiovascular risk factors.

## 2. Materials and Methods

In a cross-sectional study from March 2011 till March 2012, 1200 tile and ceramic workers were selected by simple sampling method. There were only 48 females who were excluded from the study due to low sample size. Among males, 77 subjects did not continue the study, so at last the results of physical and lab tests of 1075 workers were evaluated. Workers were selected from different parts of the factory, including grinding, ball mill, forming, glazing and printing, glaze-making, firing, packing, office, service, laboratory, maintenance, forklift driving, and warehouse.

Medical history taking and physical examination were performed by three occupational physicians who were similarly trained for this project. Demographic data (including age, gender, marital status, educational level, and job title) were recorded for each participant.

Individual or family history of CVDs was asked by the physicians. Physical examination included measuring height, weight, and blood pressure. Height was measured in standing neutral position without shoes. Weight was measured by a digital scale (Laica, China) with subjects wearing home clothing without shoes. Waist circumference was measured at 2-3 cm above the umbilicus. Hip circumference was defined as the greatest diameter between the waist and knee [18]. BMI was calculated by dividing weight (in Kg) by the square of height (in m). Blood pressure was measured after 10 minutes sitting relaxed by a mercury sphygmomanometer (Richter, Germany).

BMI was categorized according to WHO classification, that is, BMI < 18.5: underweight, 18.5 ≤ BMI ≤ 24.9: normal, BMI ≥ 25: overweight, 25 < BMI ≤ 29.9: preobesity, and BMI ≥ 30: obesity [19]. Abdominal obesity was defined as waist to hip ratio >1 in males and >0.8 in females.

Blood pressure was classified according to the guidelines of European Society of Hypertension and European Society of Cardiology, that is, SBP < 120 and DBP < 80: optimal, 120 ≤ SBP ≤ 129 and 80 ≤ DBP ≤ 84: normal, 130 ≤ SBP ≤ 139 or 85 ≤ DBP ≤ 89: high normal, and SBP ≥ 140 or DBP ≥ 90: hypertension [20].

Blood sampling was performed in the morning with at least 12-hour fasting to measure fasting blood sugar (FBS), total cholesterol (TC), triglyceride (TG), high-density lipoprotein (HDL), and low density lipoprotein (LDL). Blood parameters were analyzed by an autoanalyzer (Hitachi 902). All laboratory tests were performed in a single laboratory.

FBS level was classified based on the recommendations of the American Diabetes Association, that is, FBS < 100: desirable, 100 ≤ FBS < 125: borderline, and FBS ≥ 126: diabetes [21].

Blood lipids level was categorized according to the National Cholesterol Education Program (NCEP), that is, TG < 150: normal, 150 ≤ TG < 199: borderline, TG ≥ 200: increased. LDL < 100: optimal, 100 ≤ LDL ≤ 129: desirable, 130 ≤ LDL ≤ 159: borderline, and LDL ≥ 160: increased.

HDL ≥ 40: desirable, HDL < 40: decreased [22].

Data were analyzed by SPSS (ver. 19) using student's *t*-test and chi square test. An informed consent was obtained from each participant (in Persian). Level of significance was considered to be 0.05. This study was approved by the Ethics Committee of Shahid Sadoughi University of Medical Sciences.

## 3. Results and Discussion

### 3.1. Results

Study subjects included 1075 tile and ceramic workers. All participants were males with mean age of 31.38 ± 6.77 years. 78% of them were married.  Table 1 shows demographic data and descriptive statistics of all subjects. Ten participants (0.9%) had positive family history for CVDs and 91 participants (8.5%) were smokers.

Thirty-four participants (3.1%) had completed elementary education and 123 ones (11.5%) had completed guidance school. In addition, 871 (4.4%) and 48 (81%) participants were graduated from high school and university, respectively.

Among all subjects, 731 individuals (68%) had at least one risk factor, and 52%, 12%, 3%, and 0.7% had one, two, three, and four risk factors, respectively. Tables 2 and 3 show the frequency distribution of various risk factors.

Table 4 shows the frequency of the presence of risk factors separately in each occupational group.  Table 5 shows the anthropometric characteristics of the subjects. Tables 6 and 7 show the classification of the subjects according to BMI and blood pressure, respectively. Classification according to FBS and lipids is presented in Table 8.

### 3.2. Discussion

Cardiovascular diseases are among the most important causes of morbidity and mortality in different populations. Sedentary life style is one of the main risk factors of these diseases, so in occupational settings with low activity we expect to find a lot of risk factors, but in industrial settings in which workers are active during work shift, it seems that the frequency of CVD risk factors is low.

The main CVD risk factors include age, race, gender, smoking, hypercholesterolemia, type II diabetes, hypertension, obesity and overweight, sedentary life style, and stress [4]. Some of these risk factors are correctable and if periodically evaluated can be corrected in an early time. During periodic evaluations of workers some features of general health are evaluated as well. So, some of these risk factors can be addressed in occupational health evaluations.

In this study, we assessed the frequency of different CVD risk factors among tile and ceramic workers considering different job titles in this industry. This study identified a relatively active and young population with a high prevalence of cardiovascular risk factors.

Although overweight and obesity were not included in the general CVD risk prediction model of the Framingham Heart Study [23], the impact of body mass index on the risk of CVD incidence has been proved by many studies. European guidelines on cardiovascular disease prevention suggested that avoiding overweight or reducing the existing overweight is important in patients with established CVD as well as in high risk people [5].

In our study, overweight was observed in about half of the subjects and obesity in about 15% of them, which is very high if we consider that we have evaluated a relatively young and active population, because they were industrial workers who are completely active at least for 8 hours in their working shift, although this frequency was lower than those of Yazd urban population with 16.5% obesity [24], Brazilian workers with 68% overweight [8], bus drivers with 76.5% overweight [9], and workers of Isfahan electricity company with 60.3% overweight [25]. In other studies also overweight and obesity were CVD risk factors [26, 27], but in a study among health care workers in Singapore BMI was not an important risk factor [28].

The mean BMI in this study was similar to that in Tehran urban population (25.7%), but the frequency of overweight was lower in that study (40%) [29]; although these studies have been performed in different populations and different times, so it is possible that their risk factors are different. Abdominal obesity in this study was lower than that in general population of Yazd [24], although in the study on general population most cases of abdominal obesity were females and our study consisted of males only.

Hypertension as one of the other important risk factors of CVDs was lower in our study compared to 27.59% in Yazd adult population [24] and 20.4% in Tehran adult population [29]. This measure in tile workers was lower than those of Isfahan electricity company (16%) [25], and bus drivers in Brazil (35%) [9], and health care workers of Taiwan (29%) [15]. This low frequency of hypertension in this occupational group can be explained by their younger age compared to that of Yazd adult population, and their lower job stress compared to those of HCWs, bank workers, and managers [26, 27].

Frequency of abnormal FBS in this study was lower than those of Yazd urban population [24], Tehran general population [29], Polish managers [26], and Indian bankers [27]. This difference can also be explained by lower age and higher activity of our samples, although this measure was higher than that of HCWs in Nkombua study [30].

The frequency of smokers in this study was lower than those of Yazd urban population [24], Prabhakaran et al. study in India [31], Bugajska et al. study in Poland [26], and some other studies [8, 25, 29], although we are not sure about this issue that how much we can trust workers' self-report. In health care workers smoking was less frequent than in our population [28, 30] which can be explained by their higher social and educational level.

Mean TG level was similar to those of industrial workers in India [31] and bus drivers in Brazil [9], although it was lower than those of managers in Poland [26] and electricity company workers [25], which can be again explained by their higher level of activity.

Hypertriglyceridemia in this study was lower than those of Indian industrial workers [31], Isfahan electricity company workers [25], and Kashan drivers [32], which again can be explained by higher level of activity in this occupational group. This measure was higher in our samples compared to HCWs in Singapore [28] and Brazilian workers [8].

Other risk factors such as increased LDL and cholesterol were also different in this study from other studies on different jobs [24, 26, 27, 29, 31].

The most frequent risk factor in this study was abnormal BMI; Low HDL and increased TG were in the 2nd and 3rd orders. Overweight was most commonly observed in warehouse workers which can be explained by their lower level of activity compared to other workers. Overweight in office workers, who have sedentary work as well, was lower than in warehouse workers, which is probably due to their higher social level and different nutrition style.

## 4. Conclusions

This study showed a relatively high prevalence for CVD risk factors among tile and ceramic workers. Low HDL, high TG, and overweight were the most frequent risk factors in this population.

## Figures and Tables

**Table 1:** Demographic and descriptive data of all participants.

Variable	Mean (SD)
Age (years)	31.38 (6.77)
Height (m)	173.60 (7.36)
Weight (Kg)	76.20 (14.19)
BMI (Kg/m^2^)	25.32 (4.45)
SBP (mmHg)	112.77 (9.16)
DBP (mmHg)	71.87 (5.1)
FBS (mg/dL)	90.23 (25.32)
TC (mg/dL)	182.36 (64.23)
TG (mg/dL)	149.54 (84.49)
LDL (mg/dL)	83.59 (37.17)
HDL (mg/dL)	40.90 (9.58)

SBP: systolic blood pressure; FBS: fasting blood sugar; DBP: diastolic blood pressure; TG: triglycerides; LDL: low-density lipoprotein; HDL: high-density lipoprotein; SD: standard deviation.

**Table 2:** Frequency distribution of BMI, SBP and DBP, and FBS among all subjects.

	Classification	Cut point	No. (%)
BMI	Underweight	<18.5	62 (5.8)
	Normal	18.5–24.9	479 (44.6)
	Overweight	≤25	534 (49.6)
	Preobesity	25–29.9	376 (35.0)
	Obesity	≥30	158 (14.6)

Abdominal circumference	Normal	<1 in males	948 (88.2)
		<0.8 in females	
	Abnormal	>1 in males	127 (11.8)
		>0.8 in females	

Blood pressure	Optimal	SBP < 120	888 (82.6)
	Normal	SBP 120–129	125 (11.6)
	High normal	SBP 120–129	32 (3.0)
	Hypertension	SBP ≥ 140	30 (2.8)
	Optimal	DBP < 80	977 (90.9)
	Normal	DBP 80–84	59 (5.5)
	High normal	DBP 85–89	17 (1.6)
	Hypertension	DBP ≥ 90	22 (2.0)

Fasting blood sugar	Desirable	<100	926 (87)
	Borderline	100–125	116 (10.9)
	Increased	≥126	22 (2.1)

**Table 3:** Frequency distribution of total cholesterol (TC), triglyceride (TG), high-density lipoprotein (HDL), and low density lipoprotein (LDL) in tile workers.

Classification	Cut point	No. (%)
TG—normal	<150	652 (60.7)
TG—borderline	150–199	217 (20.2)
TG—increased	≥200	206 (19.1)

TC—normal	<200	924 (85.9)
TC—borderline	200–239	133 (12.3)
TC—increased	≥240	18 (1.8)

LDL—optimal	<100	791 (73)
LDL—desirable	100–129	229 (21.8)
LDL—borderline	130–159	44 (4.2)
LDL—increased	≥160	11 (1)

HDL—desirable	≥40	555 (51.6)
HDL—decreased	<40	520 (48.4)

**Table 4:** Frequency of the presence of risk factors in different occupational groups.

Job title	Hypertension		Overweight	Increased FBS	Increased TG	Increased LDL	Decreased HDL
	Systolic	Diastolic					
1	6.5	—	16	—	9.7	—	51.6
2	1.6	1.6	8.2	1.6	19.7	—	47.5
3	1.8	3.6	12.7	—	14.5	3.6	50.9
4	2.4	2.4	20	1.6	18.5	1.6	48.4
5	6.1	12.1	18.2	—	24.2	3	54.5
6	—	1.1	20.5	2.3	22.7	9.1	43.2
7	5.5	3	10.3	0.6	12.7	1.2	55.2
8	—	—	13	3.8	15.2	2.2	55.2
9	—	3.7	7.4	—	14.8	7.4	51.9
10	8.3	—	12.5	4.2	—	12.5	50.0
11	6.6	2.6	10.5	2.6	23.7	2	50.0
12	4.4	4.4	22.2	—	26.7	6.7	42.2
13	—	—	32.6	9.3	41.9	14	34.9

1: grinding, 2: ball mill, 3: forming, 4: glazing and printing, 5: glaze-making, 6: firing, 7: packing, 8: office, 9: service, 10: laboratory, 11: maintenance, 12: forklift driving, and 13: warehouse.

**Table 5:** Anthropometric and clinical characteristics of the population.

Variable	Number	Minimum	Maximum	Mean (SD)
Age (years)	1075	20.00	67.00	31.38 (6.77)
Height (m)	1075	139.00	198.00	173.60 (7.36)
Weight (Kg)	1075	41.00	130.00	76.20 (14.19)
BMI (Kg/m^2^)	1075	16.28	42.17	25.32 (4.45)
SBP (mmHg)	1073	75	175	112.77 (9.16)
DBP (mmHg)	1073	50	110	71.87 (5.1)
FBS (mg/dL)	1064	56	375	90.23 (25.32)
TG (mg/dL)	1066	21	739	149.54 (84.49)
LDL (mg/dL)	1051	10	657	83.59 (37.17)
HDL (mg/dL)	1059	26	174	40.90 (9.58)

SBP: systolic blood pressure; FBS: fasting blood sugar; DBP: diastolic blood pressure; TG: triglycerides; LDL: low-density lipoprotein; HDL: high-density lipoprotein; SD: standard deviation.

**Table 6:** Classification of the population based on body mass index.

Classification	Cutoff point	Number (%)
Underweight	<18.5	62 (5.8)
Normal	18.5–24.9	479 (44.6)
Overweight	25–29.9	376 (35.0)
Obesity	≥30	158 (14.6)

**Table 7:** Classification of the population based on systolic and diastolic blood pressure.

Classification	Cut point	No. (%)
Optimal	SBP < 120	886 (82.6)
Normal	SBP 120–129	125 (11.6)
High normal	SBP 120–129	32 (3.0)
Hypertension	SBP ≥ 140	30 (2.8)

Optimal	DBP < 80	975 (90.9)
Normal	DBP 80–84	59 (5.5)
High normal	DBP 85–89	17 (1.6)
Hypertension	DBP ≥ 90	22 (2.0)

**Table 8:** Classification of the population based on FBS and lipids.

Classification	Cut point	No. (%)
FBS—desirable	<100	926 (87)
FBS—borderline	100–125	116 (10.9)
FBS—increased	≥126	22 (2.1)

TG—normal	<150	643 (60.3)
TG—borderline	150–199	217 (20.4)
TG—increased	≥200	206 (19.3)

LDL—optimal	<100	767 (73)
LDL—desirable	100–129	229 (21.8)
LDL—borderline	130–159	44 (4.2)
LDL—increased	≥160	11 (1)

HDL—desirable	≥40	520 (50.9)
HDL—decreased	<40	520 (49.1)
